# Pregnancy-related sensory deficits might impair foraging in echolocating bats

**DOI:** 10.1186/s12915-023-01557-7

**Published:** 2023-03-28

**Authors:** Mor Taub, Omer Mazar, Yossi Yovel

**Affiliations:** 1 School of Zoology, Faculty of Life Sciences, Tel Aviv University, 6997801 Tel Aviv, Israel; 2 Sagol School of Neuroscience, Tel Aviv University, 6997801 Tel Aviv, Israel

**Keywords:** Echolocation, Sensing, Pregnancy, Reproduction, Movement

## Abstract

**Background:**

Reproduction entails substantial demands throughout its distinct stages. The mammalian gestation period imposes various energetic costs and movement deficits, but its effects on the sensory system are poorly understood. Bats rely heavily on active sensing, using echolocation to forage in complete darkness, or when lighting is uncertain. We examined the effects of pregnancy on bat echolocation.

**Results:**

We show that pregnant Kuhl’s pipistrelles (*Pipistrellus kuhlii*) altered their echolocation and flight behavior. Specifically, pregnant bats emitted longer echolocation signals at an ~ 15% lower rate, while flying more slowly and at a lower altitude compared to post-lactating females. A sensorimotor foraging model suggests that these changes could lead to an ~ 15% reduction in hunting performance during pregnancy.

**Conclusions:**

Sensory deficits related to pregnancy could impair foraging in echolocating bats. Our study demonstrates an additional cost of reproduction of possible relevance to other sensory modalities and organisms.

**Supplementary Information:**

The online version contains supplementary material available at 10.1186/s12915-023-01557-7.

## Background

Reproduction is physiologically demanding and entails high costs, especially for females [1–7]. The gestation period is characterized by a dramatic increase in energetic demands resulting from investment in the production of reproductive tissue and in the developing fetus [4], both of which result in increased overall body mass. The need to conserve energy during this period affects female’s activity levels [5, 7, 8], e.g., reduced wheel exercise activity in lab rats and mice [2, 8]; reduced foraging, such as diving time in elephant seals [9]; and reduced flight time in bats [7, 10]. Gestation-related morphological changes can also impair maneuverability [11] and speed [12, 13]. Pregnant bottlenose dolphins, for example, face an increase in body surface elevating drag, slowing their swimming speed [12]. Similar speed reductions have been reported in running speed in lizards [13] and in flight speed in birds [14, 15] and bats [16, 17]. Flight costs are also higher for heavier (pregnant) bats, and the added mass could affect the bats’ ability to preserve lift and flight [18].

In contrast to the effects of gestation on movement, there has been little research on its effects on echolocation. We examined the effects of pregnancy on sensing in echolocating bats. Bats are the only mammals capable of powered flight and most echolocate [19–22]. Bats expend a large part of their daily energy budget on foraging [3, 23], and echolocation allows them to maintain a positive energy budget. Very little is known about the direct effects of reproduction on echolocation in bats. Two earlier studies indirectly examined the effects of pregnancy on bats (including it as a factor in a statistical model) and reported the use of lower signal frequencies during gestation [24, 25]. A third study focused on the social context of echolocation calls in sedentary bats and found no differences in echolocation of females during the mating and non-mating seasons [26]; however, they did not address pregnancy directly. Apart from these few exceptions, most studies on echolocation have either excluded pregnant females or ignored their reproductive state, so the full effect of gestation on echolocation is unknown. We tested the hypothesis that pregnancy impairs sensing in bats. Specifically, we predicted that pregnant Kuhl’s pipistrelles would alter their echolocation and flight performance, which would negatively impact their performance.

We compared the echolocation and movement between pregnant and post-lactating *P. kuhlii*. We recorded bats in both groups while they searched for and landed on a platform in a flight room. Pregnant females altered both their echolocation and flight behavior. We then used a sensorimotor model to assess how these differences might impact prey capture by simulated bats.

## Results

**Fig. 1:**
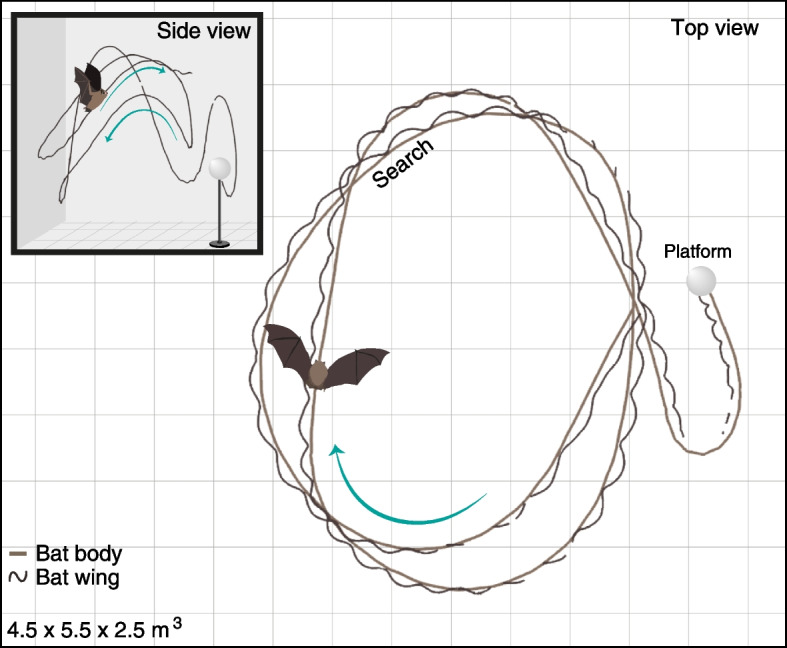
Experimental set-up and flight path. The bats flew in a 4.5 × 5.5 × 2.5-m.^3^ flight room equipped with 20 tracking cameras and 46 ultrasonic microphones spread around the circumference of the room. In each trial, the bat would fly in search of a small foam spherical landing platform, land on it, and retrieve a mealworm from the top. We tracked the bats’ movement including its center of mass and wing position and its echolocation throughout the search phase. The image shows a single search and landing event, both in a top view (showing changes in curvature) and a side view (showing changes in altitude)

We compared the echolocation and flight performance of female Kuhl’s pipistrelles during two different stages: pregnancy and post-lactation. Ten bats (five different females in each group) were trained to search for and land on a small (5-cm diameter) weakly reflecting foam platform. The platform was moved to a different location within a large 4.5 × 5.5 × 2.5-m^3^ anechoic flight room after each landing in order to ensure that the bats would continue to search for it (Fig. 1). This set-up was aimed at mimicking foraging behavior in a confined environment, while taking into consideration the limitations of the artificial setting (see the “Discussion” section).

**Fig. 2:**
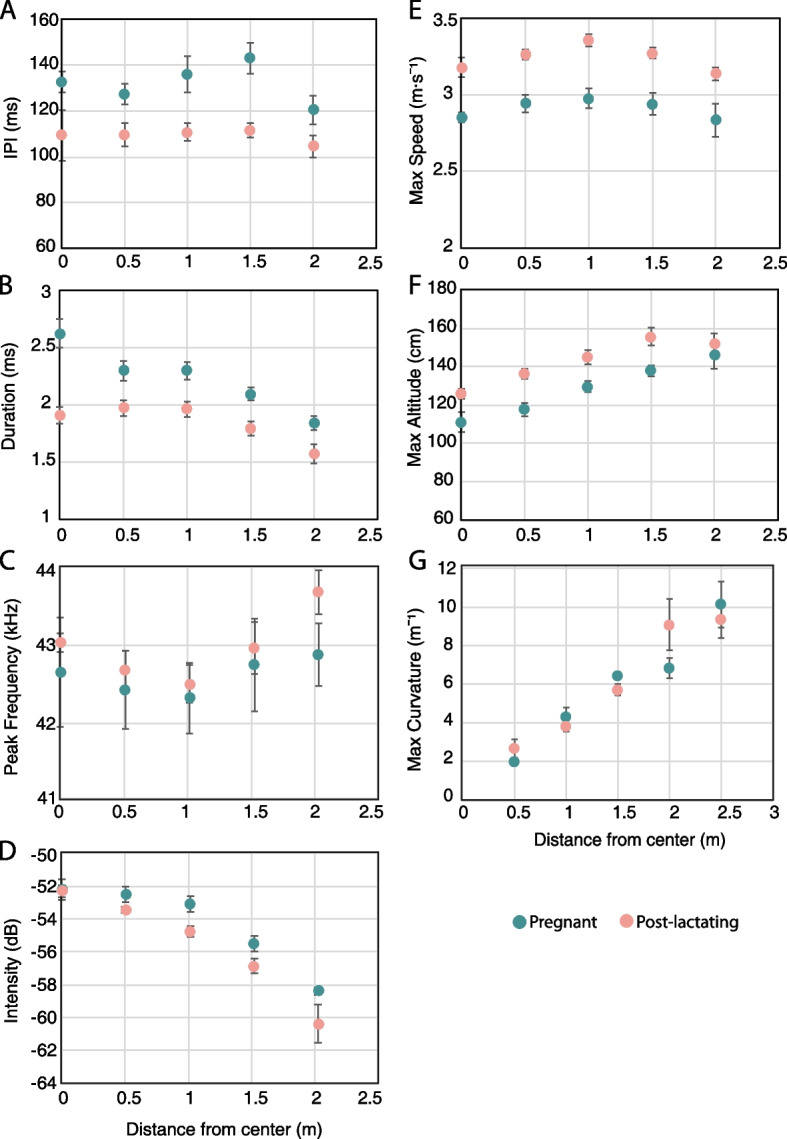
Acoustics and movement of pregnant and post-lactating bats. All parameters are presented for different distances from the center of the room (in bins of 0.5 m). For each bin, the mean ± SE is presented for *n* = 5 bats in each group. **A** Pregnant bats (green) used significantly higher inter-pulse intervals than post-lactating bats (pink). **B** Pregnant bats produced significantly longer signals. **C** There was no significant difference in peak frequency between the groups. **D** There was no significant difference in peak signal intensity between the two groups. **E** Pregnant bats flew at significantly lower (maximum) speeds throughout the search phase. **F** Pregnant bats flew at significantly lower (maximum) altitudes. The landing platform was located at ~ 110 cm above the floor. **G** There was no significant difference in (maximum) curvature of the flight path between the groups

The pregnant bats’ echolocation underwent a change in several respects: they used longer inter-pulse intervals (IPI) and longer signal durations (Fig. 2A, B; 132 ± 9 vs. 109 ± 3 ms and 2.2 ± 0.3 vs. 1.8 ± 0.2 ms (mean ± SD), *P* = 0.003 and *P* = 0.03, respectively, *n* = 10, mixed-effect GLM with the reproductive condition set as a fixed factor, the distance from the center of the room, and body mass index (BMI) as additional fixed covariates and bat ID and trial number as random effects). Note that as we include the distance from the walls as an explanatory parameter, these changes in echolocation cannot be explained by a difference in the coverage of space. The pregnant bats also exhibited some alteration to their signal frequency and intensity but the differences between the two groups were not significant (Fig. 2C, D; *P* = 0.4, *P* = 0.1, respectively, *n* = 10; mixed-effect GLM as above). There was, however, a significant effect of the bats’ body mass index (BMI) on the signal frequency (*P* = 0.02, mixed-effect GLM as above; Additional file 1: Fig. S1). We also compared the wingbeat rate of the two groups, since bats are known to adjust emission timing to their wingbeat in order to conserve energy [27, 28], but found no significant difference (*P* = 0.5, mixed-effect GLM with BMI as the fixed effect and bat ID and trial number as random effects, *n* = 10). The distribution of the IPIs between the groups indicates that the pregnant bats emitted calls once every wingbeat or every other wingbeat more frequently than the post-lactating bats (Additional file 2: Fig. S2).

Next, we compared the bats’ flight performance and found that the pregnant bats flew at lower speeds and altitudes compared to the post-lactating females. There was a significant difference between the groups in both average and maximum speed, with the pregnant bats flying on average 0.3 m/s more slowly than the post-lactating bats (Fig. 2E; 2.8 ± 0.06 vs. 3.1 ± 0.04 m/s (mean ± SD), *P* = 0.005 and *P* = 0.004 respectively, *n* = 10; mixed-effect GLM as above). In addition, the pregnant bats flew at a lower height. Although there was no significant change in the average flight altitude between the two groups (mixed-effect GLM as above, *P* = 0.06, *n* = 10), the maximum altitude reached by the pregnant bats was significantly lower (Fig. 2F; 128 ± 14 vs. 143 ± 12 cm (mean ± SD), *P* = 0.03, *n* = 10; mixed-effect GLM as above). We also tested the bats’ horizontal maneuverability and found no significant difference in flight curvature between the two groups (Fig. 2G; *P* = 0.5, *n* = 10; mixed-effect GLM as above).

**Fig. 3:**
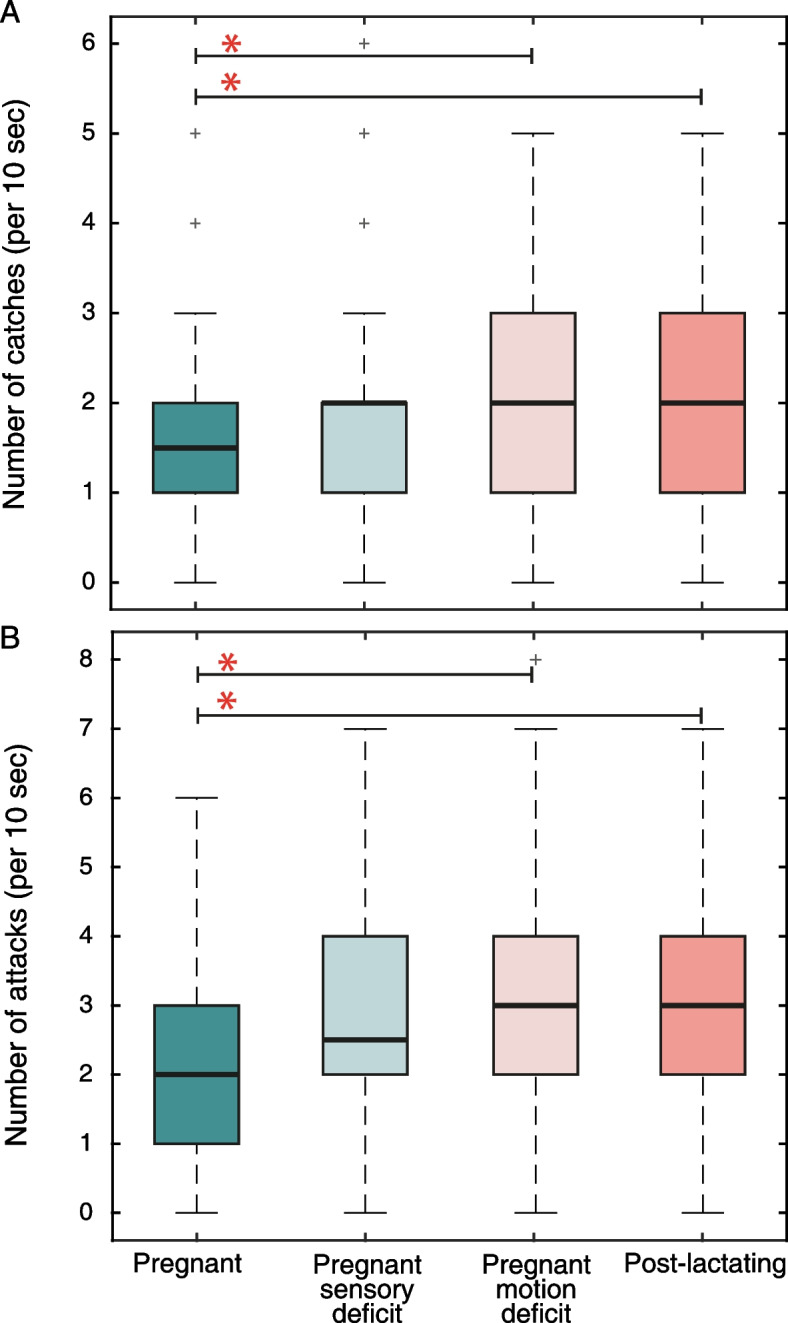
Sensorimotor foraging simulation of the different reproductive conditions. All panels show box plots where whiskers indicate the extreme data points (beyond the percentiles) that are not considered outliers, and plus signs indicate outliers. Asterisks indicate a significant difference between the relevant groups. **A** The simulated pregnant bats caught prey at a lower rate than the post-lactating and pregnant-motion-deficit bats. **B** The simulated pregnant bats attacked prey at a lower rate than the post-lactating and pregnant-motion-deficit bats. There was no difference between the pregnant-sensory-deficit bats and any of the other groups in both parameters

Finally, in order to understand how these differences in sensory and flight behavior during gestation might affect hunting success, we ran a simulation mimicking bats’ sensorimotor decisions while foraging in a confined space (Additional file 3: Movie S1). We compared four groups of bats: the two experimental groups (pregnant and post-lactating), which differed in both their sensory and motor parameters, and two additional control groups that only differed in one aspect from the original pregnant group (pregnant-sensory-deficit or pregnant-motion-deficit; see the “Methods” section). This comparison allowed us to separate the sensory and motor effects of pregnancy. In the simulations, a single bat from one of the four groups, each foraged (for simulated moths) in a 10 × 10-m^2^ area. We found that the simulated pregnant bats had a significantly lower prey capture success rate than both post-lactating and pregnant-motion-deficit bats, catching 1.6 ± 1.1 vs. 1.9 ± 1.2 vs. 1.9 ± 1.1 (mean ± SD) prey items per 10 s, respectively (Fig. 3; one-way ANOVA, *P* = 0.0025, *F* = 4.8, df = 796, with Tukey–Kramer post hoc, *P* = 0.004 and *P* = 0.007 for the pregnant vs. post-lactating and pregnant vs. pregnant-motion-deficit bats, respectively). To determine the cause of the reduced performance, we examined the females’ attack rate and attack success. The simulated pregnant females attacked fewer prey items than the post-lactating and the pregnant-motion-deficit groups (2.3 ± 1.4 vs. 3.0 ± 1.6 vs. 3.0 ± 1.6 (mean ± SD) attacks per 10 s, respectively, one-way ANOVA, *P* < 0.0001, *F* = 8.9, df = 796, with post hoc as above, *P* = 0.0002 and *P* < 0.0001). In contrast, there was no significant difference in the attack success rate between the groups, i.e., in the percentage of attack attempts that resulted in capture (66 ± 28 vs. 66 ± 32 vs. 67 ± 29 vs. 67 ± 29% successful attacks, for pregnant, pregnant-sensory-deficit, pregnant-motion-deficit, and post-lactating bats, respectively, *P* = 0.72, df = 685). In all of the above tests, there was no significant difference between the post-lactating and pregnant-motion-deficit groups, suggesting that the reduced speed of pregnant females is not in itself enough to explain the change in hunting performance. Moreover, the control group of pregnant-sensory-deficit bats did not significantly differ from any of the other groups in the performed tests.

## Discussion

The mammalian gestation period has been shown to be costly on many levels [1–7]. We tested the hypothesis that gestation also impairs echolocation. We found that, during pregnancy, female bats exhibited changes to both their flight and sensory performance.

The most profound changes in echolocation were in the temporal domain: increased signal duration and reduced repetition rate. Both changes are typical for bats flying in a less cluttered (i.e., less complex) environment and imply that relative to their complex surroundings the pregnant bats were under-sampling it. Previous studies have found a correlation between pregnancy and the use of lower-frequency signals [24, 25]. We found a significant correlation only between frequency and BMI, but since the largest bats were also pregnant, this finding could support the reduction in frequency previously observed in gestating bats. However, a decrease in frequency has also been associated with seasonal changes and lower body temperature in winter [29, 30].

Why do female bats reduce their calling rate? Emitting echolocation vocalizations requires a strong pressure generated in the abdomen. Among mammals, bats are known to bear large neonates relative to the mother’s body size [31, 32]. *Pipistrellus kuhlii* females give birth to pups (often two) weighing approximately 1 g, which accounts for ~ 15% of the mother’s body weight [33]. Changes in BMI, and specifically the mass accumulated in the abdomen during gestation, could impair the mother’s ability to generate the necessary pressure, or increase its cost [34, 35]. Future studies should examine the different stages of pregnancy in order to further understand whether these costs become more profound as pregnancy advances or with different embryonic loads.

The pregnant bats also altered their flight, demonstrating a reduction in flight speed compared to the post-lactating females, a finding that has been previously associated with increased mass [17, 18]. The pregnant bats also flew at lower altitudes, closer to the height of the landing platform, and often landed below the top part of the sphere. Such reduced flight speed could have led to difficulty in maintaining lift, resulting in the observed lower flight altitude. In addition, the added size and weight of the fetus(es) may have altered the center of mass the bats experienced, making it more difficult to land precisely on a target. Pregnant women are known to experience changes in balance that increase their likelihood of falling [36, 37]. It is possible that the pregnant bats (especially the heavier ones) preferred to fly lower and closer to the height of the platform, in order to facilitate landing and reduce the need for vertical maneuvering.

Our comprehensive sensorimotor simulations reveal that the foraging performance of bats is expected to deteriorate as a result of pregnancy-induced changes, especially due to sensory deficits. Our simulations suggest that the ~ 15% lower sensory-update rates exhibited by the pregnant females will result in an ~ 15% decrease in insect detection and attack, thereby reducing the capture rate. Although the simulated pregnant bats initiated fewer attacks, there was no decrease in capture success once an attack had been initiated, suggesting that foraging is impaired due to sensory deficits rather than movement deficits.

Our findings could imply that during gestation female bats may have to alter their dietary composition and perhaps also become less selective, e.g., focus on larger and slower prey. Field studies have indeed found differences in the diet of pregnant female bats [24, 38, 39]. It was shown that during pregnancy *Rhinolophus ferrumequinum* fed mostly on moths and then added beetles to their diet while lactating [38]. One suggested explanation given for this observation was that the extra weight in late pregnancy reduced flight speed and diminished the bats’ chances of successful capture of faster prey items (i.e., beetles). Additional explanations could relate to variation in emergence time [40], affecting the availability of certain prey; or selection of different foraging sites. Our findings suggest that there could also be a sensory component contributing to the altered prey selection exhibited by the pregnant females, such as a shift to larger prey that are more easily detected.

While there are many variables that could impact foraging, such as prey availability and weather conditions, echolocation and flight performance play an important role in foraging success. In the wild, *P. kuhlii* catch their prey in flight. In this study, the bats performed a landing task in a confined space, which has different requirements and imposes certain restrictions, such as limiting flight speed. Nonetheless, the bats in our experiment exhibited behavior similar to their natural hunting behavior, making sharp turns and maneuvers and initiating approaches towards the target. Taking into account the limited space of the set-up, the flight speed demonstrated by our experimental bats did not differ greatly from the foraging and maneuvering speeds reported for this species in the wild (1.5–3.9 m/s vs. 1.8–7.5 m/s respectively) [41, 42].

## Conclusions

Little is known about the effects of pregnancy on sensing in mammals. In this study, we reveal an additional impact of gestation on echolocation in bats. Our findings suggest that pregnancy imposes a sensory cost that may negatively impact the bats’ foraging behavior. The deficits that we observed in sensing are mostly relevant for animals that rely on active sensing, which requires muscular activity such as sound emission. Indeed, many sensory systems rely on muscular activity, such as focusing depth of field using vision, ear movement, sniffing and whisking, which might become impaired during pregnancy.

## Methods

### Animals

Eleven *Pipistrellus kuhlii* bats (five post-lactating females and six pregnant females) were captured under a permit from the Israeli National Park Authority (permit number 2016/41421). Post-lactating females were captured at the very end of the lactation season (late August), after the pups had been weaned and prior to the following mating season [43], ensuring that they were not pregnant. Pregnant females were captured in April and underwent an ultrasound scan to validate their condition both upon arrival and prior to release. *P. kuhlii* bats often give birth to twins, and within our group of six bats at least four of the females were pregnant with twins, as revealed in the ultrasound (one bat had a single embryo and another bat was in too early a stage of pregnancy to evaluate the number of embryos it was carrying). During the experiment, one of the bats miscarried a single embryo, but we were unable to determine which bat it was. A second ultrasound performed after the end of trials confirmed that all five bats still had at least one embryo. One pregnant female gave birth before completing all trials and was excluded from the experiment. The bats were housed at Tel Aviv University’s Zoological Gardens under a reversed light–dark cycle and a temperature of 23–26 °C. The experimental protocols and procedures were approved and performed according to the Institutional Animal Care and Use Committee of the Israeli Health Ministry (ethics approval number: 04–18-026).

### Experimental setup and design

The experiment took place in a 4.5 × 5.5 × 2.5-m^3^ sound-isolated flight room with acoustic foam on the walls and ceiling. The bats were trained in this room to land in the dark on a spherical platform positioned in the center of the room (110 cm above the ground), where mealworms were offered. The bats were first trained to land on a 15-cm diameter Styrofoam sphere and later on a 5-cm diameter weakly reflecting foam sphere that served for data collection. This latter sphere was intended to create a target that was difficult to detect. During the experiment, the platform was moved to a different location in the room after each landing, alternating occasionally between the large Styrofoam sphere and the small foam sphere. We analyzed the flight and echolocation of both groups (pregnant and post-lactating) while searching for the small foam platform. The pregnant bats took on average 114 ± 106 (mean ± SD) seconds to land on the small platform for the first time, while the post-lactating bats took 44 ± 15 (mean ± SD) seconds to the first landing. The difference between the groups was not significant (*t*-test, *P* = 0.2) and was possibly affected by individual variation, since only two of the five pregnant bats searched for longer than 50 s (216 and 242 s). In order to account for differences in mass between the pregnant and post-lactating females, we measured their forearm length upon capture and weighed them once a week to calculate each bat’s body mass index (BMI; see Additional file 4: Table S1). There was no significant difference between the BMIs of the two groups (*t*-test, *P* = 0.13), but one of the pregnant bats (bat no. 4) was in the early stages of pregnancy (as revealed by the ultrasound) and weighed substantially less than the rest of the group (Additional file 4: Table S1). Reassessing the BMI, while excluding this bat, resulted in a significant difference between the groups (*t*-test, *P* = 0.007). We included the BMI as a covariate in all statistical analyses to test for its possible effect on the other parameters. In addition, since the bats flew in an enclosed room in which both their echolocation and movement might be affected by their proximity to the walls, we also took into consideration the bats’ spatial location relative to the center of the room (there was no significant difference in distances between the groups, mixed-effect generalized linear model (GLM) with the reproductive condition set as a fixed factor, BMI as covariate, and bat ID and trial no. as random effects, *P* = 0.3, *n* = 10).

### Tracking, video, and audio recordings

Tracking was performed using a Motion Analysis Corp system. Twenty cameras (16 Raptor cameras, 1280 × 1024 pixels, and 4 Raptor 12 cameras, 4096 × 3072 pixels) were used to track the bats at a frame rate of 200 fps. Two spherical reflectors (2.4-mm diameter, 3X3 Designs Corp.) were attached to the bats using double-sided tape. One reflector was mounted between the shoulder blades and the other was placed on the wing, allowing us to track both center of mass and wingbeat. Previous experiments had confirmed that this system is able to track a moving reflector to an accuracy of ~ 1 mm [44]. Audio recordings were performed using 46 ultrasonic wide-band microphones (USG Electret Ultrasound Microphones—Avisoft Bioacoustics/Knowles FG) connected to four Hm1216 AD converters sampling at a rate of 375,000 Hz. All channels were synchronized by injecting an SMPTE code (Horita) into the least significant bit of their first channel. The microphones were evenly spread around the circumference of the room (100 cm between each two microphones) at a height of 60 cm, 120 cm, or 180 cm. The audio recordings were synchronized to the video tracking (Motion Analysis, Inc.).

### Audio analysis

Echolocation parameter extraction was performed in Batalef: a MATLAB-based in-house software created for acoustic analysis [45, 46]. For each landing event, we analyzed an ~ 2-s segment from the search phase of the echolocation sequence. Signals were detected automatically and then manually scrutinized to remove false detections. Four parameters were extracted from each signal: signal duration (defined according to a decrease of − 12 dB relative to the peak); peak intensity; inter-pulse interval (defined as the time between the start of one pulse and the start of the consecutive pulse); and peak frequency (frequency with most energy). Except for the frequency, all echolocation parameters were measured from the envelope of the time signal. Since recordings were performed using 46 microphones, for each signal in the sequence, we selected the channel with the highest intensity and used the data extracted from that channel for all analyses. The peak intensity was normalized according to the bat’s distance from the selected microphone.

### Behavioral analysis

For each search flight, we measured the speed, altitude, and curvature derived from the flight path recorded using the tracking system. The wingbeat rate was measured from the tracking of the wing. We recorded a total of 292 flights ending in a landing (165 for the pregnant bats and 127 for the post-lactating bats).

### Sensorimotor simulation

A MATLAB-based model, recently developed in our lab, was used to simulate the flight and echolocation of pregnant and post-lactating *P. kuhlii* bats to test for differences in prey capture success. The model simulated the flight of the prey (exhibiting moth movement characteristics, flying without any response to the bats), as well as the bat’s sensory and flight behavior, and received acoustic input (echoes returning from the prey; see Mazar and Yovel [47] for a comprehensive description of the model and source code, and see Additional file 3: Movie S1 for an example of one simulation). The model simulated the flight of a single bat in a 10 × 10-m^2^ 2D area with three moths at a time and no obstacles (aside from the borders of the area). We modeled the echolocation behavior and movement of each group based on the data collected from the search phase during this study. We adjusted four parameters according to the group: signal duration (2.4, 2.0 ms for pregnant and post-lactating, respectively); peak frequency (43, 45 kHz); inter-pulse interval (130, 110 ms); and flight speed (2.7, 3.1 m/s). In order to determine whether the changes in performances were influenced by a sensory deficit or a movement deficit, we added two control groups that exhibited only one of the deficits exhibited by the pregnant bats—either the echolocation (pregnant-sensory-deficit) or the movement (pregnant-motion-deficit). Once a target was detected by the simulated bats, the approach phase was identical for all groups, but it began with longer intervals and durations for the pregnant bats reflecting their behavior in reality. The attack success rate was defined as the total number of captures divided by the number of initiated approach sequences. Each scenario, in which a single bat is hunting for prey, was simulated 200 times for each of the four types of bat.

### Statistics

To test for changes in echolocation and flight parameters in the pregnant bats, we used a mixed-effect GLM model with the reproductive condition set as a fixed factor, bat ID and trial number as random effects, and distance from the center of the room and BMI (weight/(forearm^2^)) as covariates. We did not add interactions between variables after comparing the Bayesian information criteria (BIC) of the two models (with and without interactions), and asserting that the model used here showed a better fit. We tested an additional model that included the effect of the forearm length but again found that the BIC of the original model (without forearm length) showed a better fit. The analysis was performed on the maximum or average of each parameter within distance bins ranging from 0 to 3 m (in 0.5-m increments). To test for differences between the groups in the simulation, we performed one-way ANOVA, with the reproductive condition set as the fixed factor. We then used a Tukey–Kramer post hoc multi-comparison to test the effect of each group (see Additional file 5: Table S2 for all statistical results). All analyses were performed in MATLAB.

## Supplementary Information

**Additional file 1:** **Fig. S1**. Acoustic and movement parameters by BMI. There was a significant difference in BMI between groups (t-test, P=0.007; with the exclusion of the smallest bat from the pregnant group) and no significant effect of BMI on any of the parameters except for peak frequency. Each point depicts the mean±SE of a single bat from each group (pink: post-lactating; green: pregnant). **(A)** There was a difference in IPI between the two groups regardless of the bats’ BMI. **(B)** The difference in signal duration was not dependent on BMI. **(C)** Signal intensity was not affected by BMI. **(D)** Maximum speed was not affected by BMI and differed between the groups. **(E)** Flight altitude seemed to have decreased with increase in mass for the largest bats in the group, but was not significant. **(F)** There was no difference in curvature with increase in mass. **(G) **There was an effect of BMI on the peak frequency (GLM, P=0.02).

**Additional file 2:** **Fig. S2.** IPI distribution of the two reproductive conditions. The pregnant bats (green) emitted calls once every wingbeat or every second wingbeat (see black arrows) more often than the post-lactating bats (pink). Lines represent mean values and shaded areas represent the SE. N=5 for each group.

**Additional file 3:** **Movie S1.** Sensorimotor simulation of a single bat. The upper panel shows a simulated bat (blue) hunting three prey items (pink, magenta, and purple). Green circles indicate the bat’s detection and localization of the objects (either the prey or the walls). The lower panel depicts the bat’s echolocation signals (signal level as a function of time): transmitted calls (black), echoes reflected from the prey (green), and echoes reflected from the walls (pink). Red 'x' depicts a collision with a wall and blue '+' depicts a successful capture.

**Additional file 4:** **Table S1**. Body measurements of the bats in the two reproductive conditions. The body-mass index was calculated by dividing each bat’s average weight by the square of its forearm length.

**Additional file 5:** **Table S2.** Statistical P-values for the different acoustic and movement parameters. Three effects were tested: the reproductive condition (pregnant vs. post-lactating), the body-mass index (BMI); and the bats’ distance from the center of the room. For sensorimotor model statistics, results are shown for the comparison of all four groups: 1. Pregnant; 2. Pregnant-sensory-deficit; 3. pregnant-motion-deficit; 4. Post-lactating and the relevant between-groups. Significance below 0.05 is marked in red.

## Data Availability

The datasets generated and analyzed during the current study are available on Mendeley Data: http://doi.org/10.17632/hbb2t3dnbc.1 (48).

## Abbreviations

BMI: Body mass index
IPI: Inter-pulse interval
