# Supplementary figures and images for: Pregnancy-related sensory deficits might impair foraging in echolocating bats
Source: BMC Biol. 2023 Mar 28;21:60. doi: 10.1186/s12915-023-01557-7 (PMC10044376; doi:10.1186/s12915-023-01557-7)

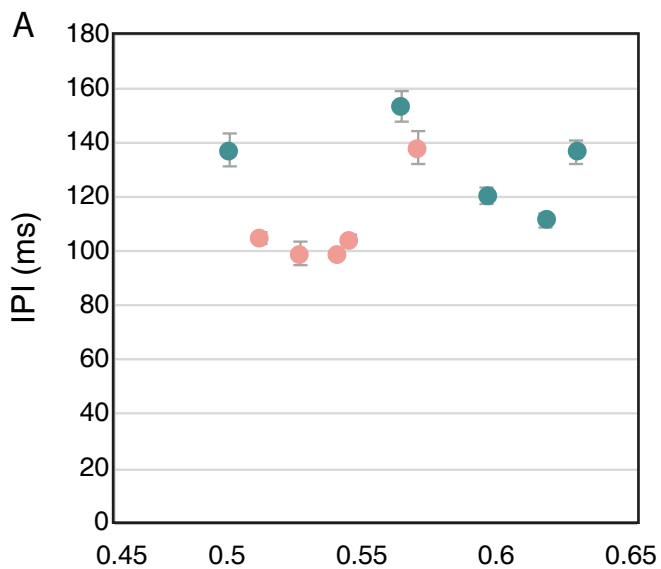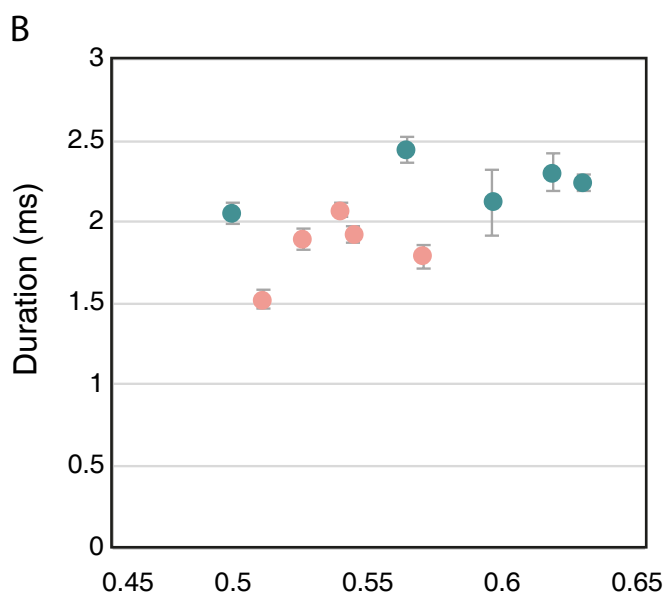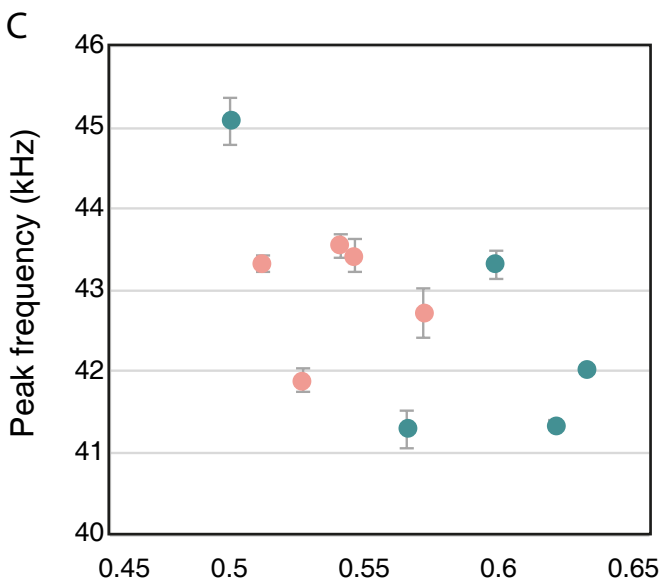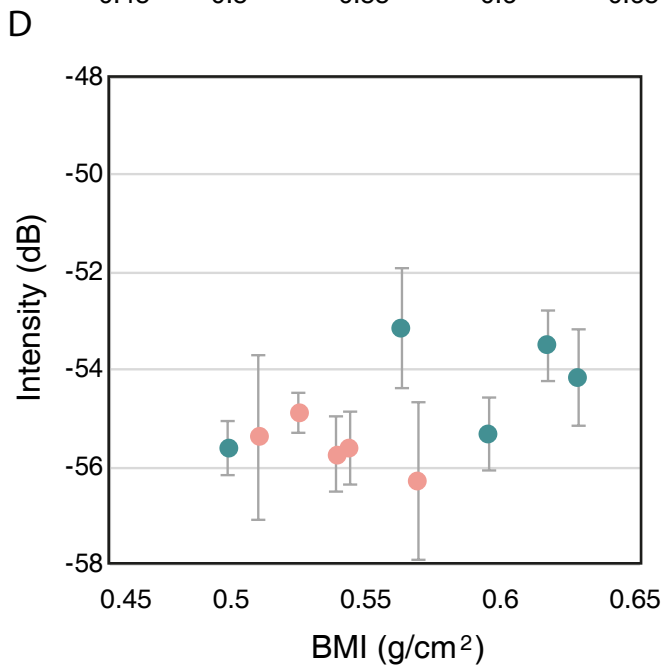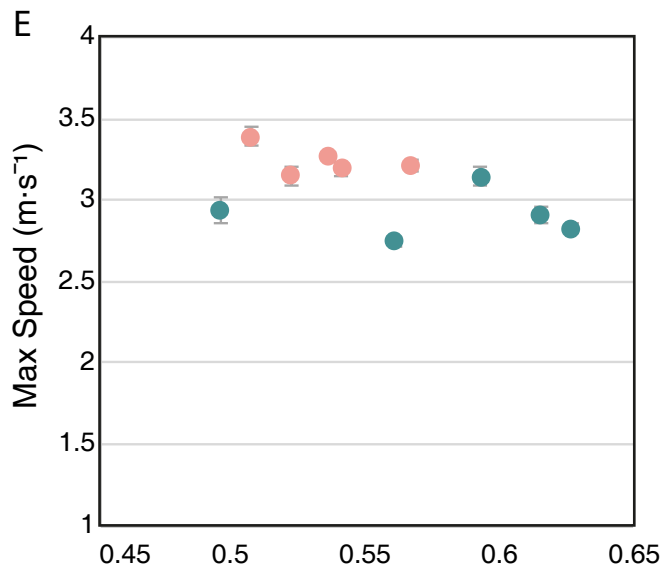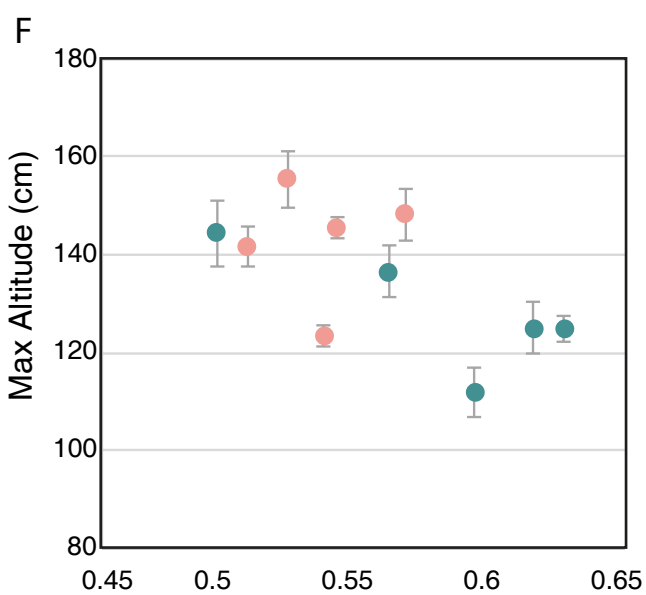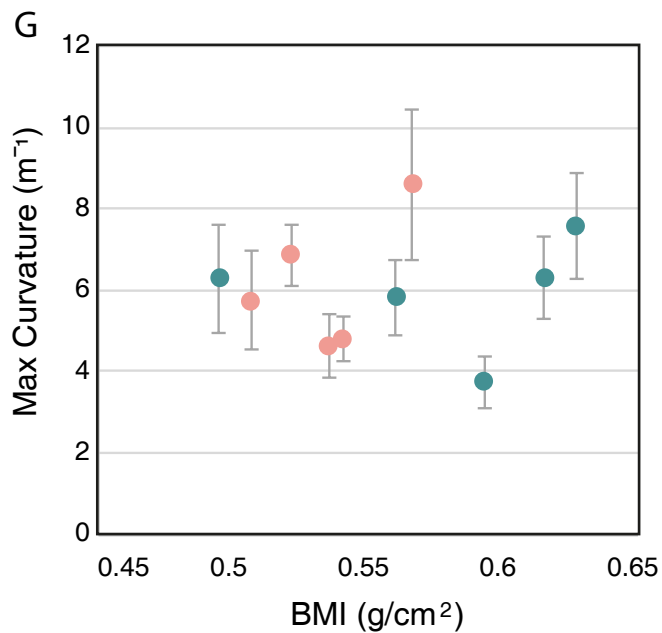

● Pregnant ● Post-lactating

Supplement: Supplementary file 1 — Additional file 1: Fig. S1. Acoustic and movement parameters by BMI. There was a significant difference in BMI between groups (t-test, P=0.007; with the exclusion of the smallest bat from the pregnant group) and no significant effect of BMI on any of the parameters except for peak frequency. Each point depicts the mean±SE of a single bat from each group (pink: post-lactating; green: pregnant). (A) There was a difference in IPI between the two groups regardless of the bats’ BMI. (B) The difference in signal duration was not dependent on BMI. (C) Signal intensity was not affected by BMI. (D) Maximum speed was not affected by BMI and differed between the groups. (E) Flight altitude seemed to have decreased with increase in mass for the largest bats in the group, but was not significant. (F) There was no difference in curvature with increase in mass. (G) There was an effect of BMI on the peak frequency (GLM, P=0.02). [file 12915_2023_1557_MOESM1_ESM.pdf]

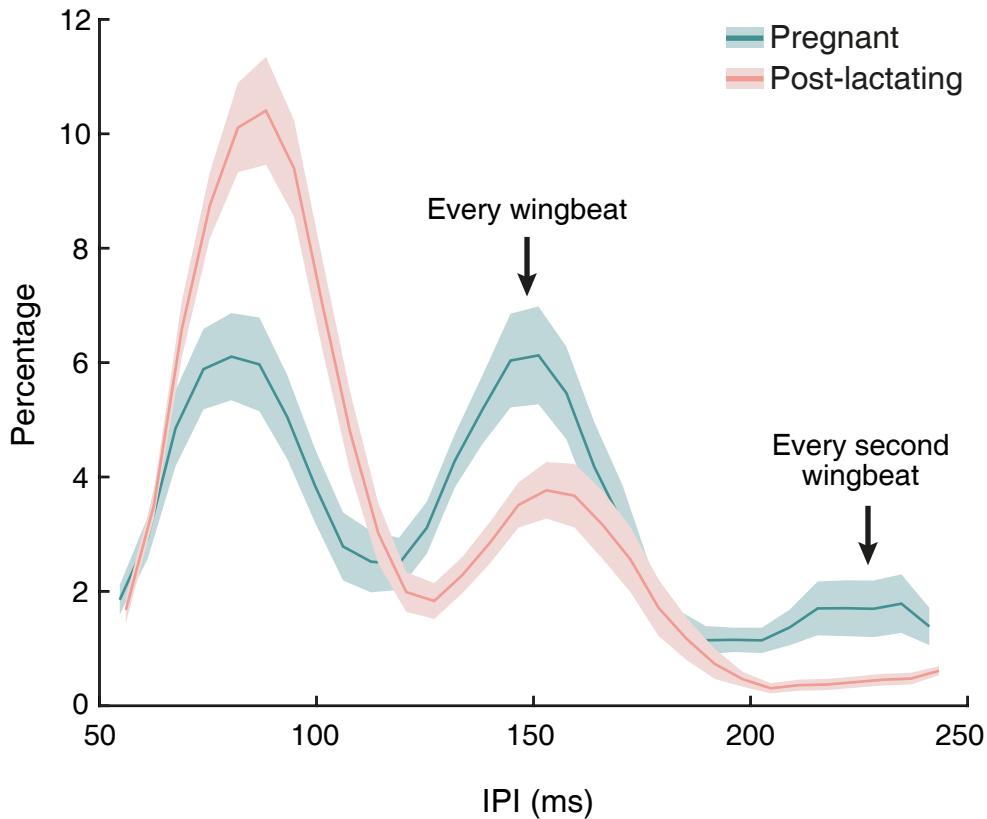

Supplement: Supplementary file 2 — Additional file 2: Fig. S2. IPI distribution of the two reproductive conditions. The pregnant bats (green) emitted calls once every wingbeat or every second wingbeat (see black arrows) more often than the post-lactating bats (pink). Lines represent mean values and shaded areas represent the SE. N=5 for each group. [file 12915_2023_1557_MOESM2_ESM.pdf]
