# Supplementary material for: Pregnancy-related sensory deficits might impair foraging in echolocating bats
Source: BMC Biol. 2023 Mar 28;21:60. doi: 10.1186/s12915-023-01557-7 (PMC10044376; doi:10.1186/s12915-023-01557-7)
Supplement: Supplementary file 4 — Additional file 4: Table S1. Body measurements of the bats in the two reproductive conditions. The body-mass index was calculated by dividing each bat’s average weight by the square of its forearm length. [file 12915_2023_1557_MOESM4_ESM.pdf]

Bat	Reproductive Condition	BMI (g/mm^2)	Average weight (g)	Forearm length (mm)
1	Pregnant	0.0062	7.54	35
2	Pregnant	0.0059	7.26	35
3	Pregnant	0.0056	6.86	35
4	Pregnant	0.0049	6.23	35.5
5	Pregnant	0.0063	6.83	33
6	Post-lactating	0.0054	6.94	36
7	Post-lactating	0.0057	7.02	35.2
8	Post-lactating	0.0052	6.60	35.6
9	Post-lactating	0.0051	6.41	35.6
10	Post-lactating	0.0054	6.58	34.9
