# Supplementary material for: Pregnancy-related sensory deficits might impair foraging in echolocating bats
Source: BMC Biol. 2023 Mar 28;21:60. doi: 10.1186/s12915-023-01557-7 (PMC10044376; doi:10.1186/s12915-023-01557-7)
Supplement: Supplementary file 5 — Additional file 5: Table S2. Statistical P-values for the different acoustic and movement parameters. Three effects were tested: the reproductive condition (pregnant vs. post-lactating), the body-mass index (BMI); and the bats’ distance from the center of the room. For sensorimotor model statistics, results are shown for the comparison of all four groups: 1. Pregnant; 2. Pregnant-sensory-deficit; 3. pregnant-motion-deficit; 4. Post-lactating and the relevant between-groups. Significance below 0.05 is marked in red. [file 12915_2023_1557_MOESM5_ESM.pdf]

	Parameter/effect	Reproductive Condition	BMI		Distance from center	
**Sensing and movement (GLM)**	Average IPI	0.003	0.3		0.02	
	Average Duration	0.04	0.5		<.001	
	Average peak frequency	0.4	0.02		<.001	
	Average Intensity	0.1	0.5		<.001	
	Max Speed	0.004	0.5		0.01	
	Average Speed	0.005	0.55		<.001	
	Max Altitude	0.03	0.15		<.001	
	Average Altitude	0.06	0.2		<.001	
	Max Curvature	0.56	0.2		<.001	
	Average Curvature	0.55	0.5		<.001	
**General**	Distance from center (GLM)	0.32	0.002			
	BMI all bats (t-test)	0.13				
	BMI without bat 4 (t-test)	0.007				
		**General**	**1-3**	**1-4**	**2-3**	**2-4**
**Model (one-way ANOVA with Tukey-Kramer post hoc)**	Capture success	0.0025	0.007	0.004	0.8	0.7
	Attack rate	<0.0001	<0.0001	0.0002	0.05	0.1
	Attack success	0.72				
